# Quantum correlation enhanced super-resolution localization microscopy enabled by a fibre bundle camera

**DOI:** 10.1038/ncomms14786

**Published:** 2017-03-13

**Authors:** Yonatan Israel, Ron Tenne, Dan Oron, Yaron Silberberg

**Affiliations:** 1 Department of Physics of Complex Systems, Weizmann Institute of Science, Rehovot 76100, Israel

## Abstract

Despite advances in low-light-level detection, single-photon methods such as photon correlation have rarely been used in the context of imaging. The few demonstrations, for example of subdiffraction-limited imaging utilizing quantum statistics of photons, have remained in the realm of proof-of-principle demonstrations. This is primarily due to a combination of low values of fill factors, quantum efficiencies, frame rates and signal-to-noise characteristic of most available single-photon sensitive imaging detectors. Here we describe an imaging device based on a fibre bundle coupled to single-photon avalanche detectors that combines a large fill factor, a high quantum efficiency, a low noise and scalable architecture. Our device enables localization-based super-resolution microscopy in a non-sparse non-stationary scene, utilizing information on the number of active emitters, as gathered from non-classical photon statistics.

## Abstract (web summary)

Classical physics enabled subdiffraction-limited imaging has rarely been extended to the quantum regime. Here, Israel *et al*. develop a super-resolution localization microscopy based on non-classical photon statistics, enabling optical tracking of multiple quantum emitters.

Far-field optical microscopy, an important workhorse in biological research, is fundamentally limited by diffraction, as was established by Abbe^1^ and Rayleigh^2^. The attainable resolution is therefore limited to approximately half the wavelength of light. In the past two decades, several successful schemes to overcome the diffraction limit in microscopy were developed^3^,^4^,^5^,^6^,^7^. Many of these utilize the concept of precise localization of single emitters in a time series of sparse frames^8^. One inherent problem of these methods is the sparsity requirement, that is, a single emitter per diffraction limited spot per frame at most, slowing down the acquisition of super-resolved images^9^. Several schemes for localizing multiple emitters have been already presented^10^,^11^,^12^,^13^; however, these algorithms yield limited performance and lack robustness^14^,^15^. As shown in this work, gathering additional information on the number of active emitters, namely, photon correlation statistics, enables localization in non-sparse scenes.

In the past few years, the use of non-classical photon statistics for subdiffraction-limited imaging has been theoretically studied^16^,^17^,^18^ and demonstrated by photon correlation measurement in both a widefield^19^ and a confocal^20^ imaging geometries. In practice, however, both realizations do not exhibit a viable pathway for super-resolution imaging or particle tracking. Here we propose and demonstrate a method that rather than using photon correlation information directly, utilizes it for multi-emitter localization in a time-dependent scene. By analysing both the simultaneous detections of photons and spatial information, one can accurately determine the number of emitters contributing to an image^21^,^22^ and localize them. In contrast with optimization-based schemes^11^,^13^, here experimental information that was previously unavailable is provided as input for the localization algorithm.

## Results

### Principle

A key ingredient required for realization of this scheme is a fast, low-noise, single-photon sensitive imaging detector. Over the past two decades, progress in the technology of low-light-level sensitive cameras was an important enabling factor in the development of super-resolution microscopy techniques^12^,^23^. Still, they are quite noisy, and the frame rate of such cameras is limited to ∼1 kHz (ref. 24), washing out information contained at higher temporal frequencies. Alternative detectors based on integrated single-photon avalanche detector (SPAD) arrays on a chip typically suffer from very low fill factors, even when using microlenses^25^.

**Figure 1:**
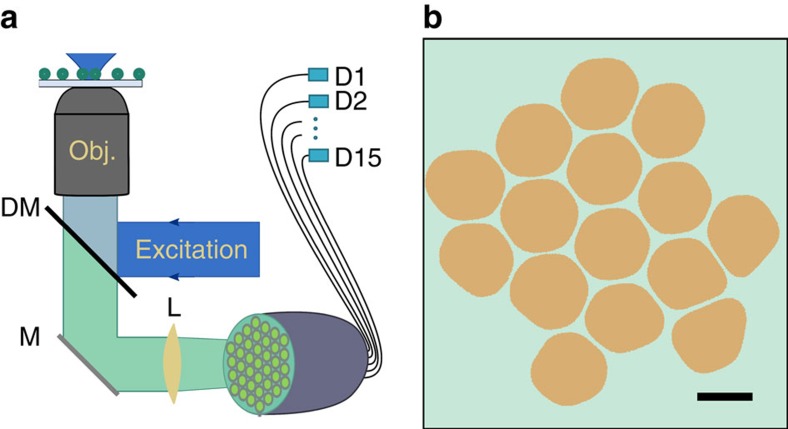
Measuring quantum correlations in a confocal microscope. (**a**) Schematic of a single-photon fibre bundle camera (SFICAM) with 15 single-photon avalanche detectors (SPADs). (**b**) Cross-section of the fibre bundle. This segmented image was compiled by thresholding an array of optical microscope images in which light was input into one of the fibres (see more details in Supplementary Fig. 5). Scale bar, 100 μm.

Our imaging device, the single-photon fibre bundle camera, is a low pixel number camera, constructed from a fibre bundle, in which each fibre acts as a pixel and guides photons to a SPAD, as shown in Fig. 1 (for more details see Methods). This device combines spatial information with a single-photon sensitivity and nanosecond scale temporal resolution, capable of detecting emission transients orders of magnitude faster than the 1 ms temporal resolution of typical cameras. Since the detectors are separated from the imaging facet of the bundle, a fill factor of over 80% is achieved (see Supplementary Note 1 and Supplementary Fig. 1). These characteristics allow us to efficiently analyse quantum photon–photon correlations within an image.

Many fluorophores are inherently single-photon emitters, for example, dye molecules and quantum dots (QDs)^26^. Therefore, simultaneously detected pairs of photons from such fluorophores provide valuable information concerning the number of emitters in every frame. Supposing that *n* identical single-photon emitters are measured, their zero delay (*τ*=0) second-order photon correlation^27^ (*g*^(2)^) will be


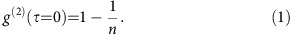


By measuring quantum correlations, the number of active emitters can be found, as seen from equation (1). In particular, it can determined from such measurements whether only a single emitter is switched on in the detection volume. We therefore continuously evaluate *g*^(2)^(0) to estimate the number of emitters contributing to an image at every point along the acquisition time. Finally, a localization algorithm can be applied to localize the emitters, using the precise number of emitters in the image.

### Single emitters

**Figure 2:**
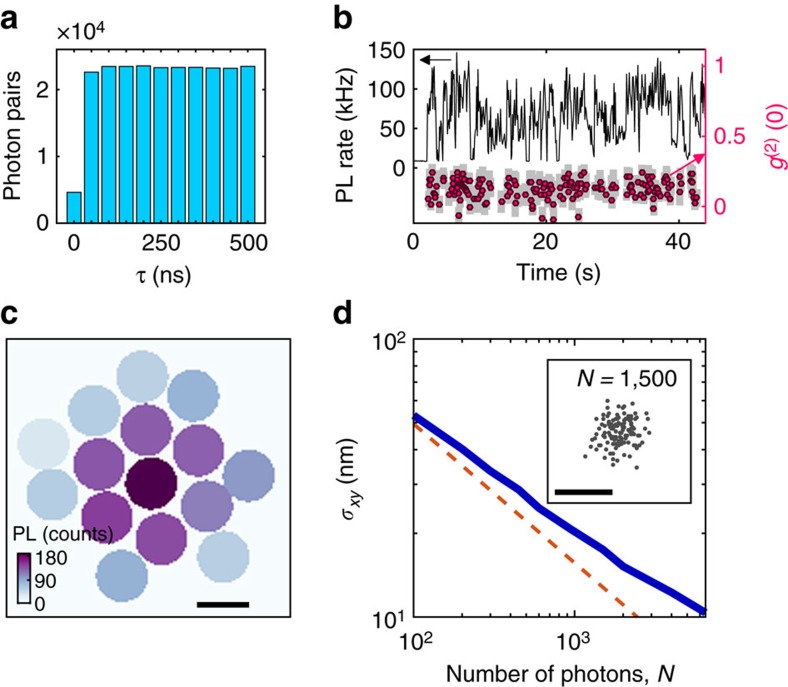
Localization of a single emitter using a fibre bundle. (**a**) Two-photon correlation count and (**b**) photoluminescence (PL) count rate (black line), summed over all detectors, and quantum correlation (red dots), summed over all detector pairs, for a single quantum dot (QD). Error bars represent ±*σ* statistical error (grey). (**c**) A single QD imaged by the bundle camera using *N*=1,500 photons acquired in 15 ms. Colour bar represent PL count. Scale bar, 100 μm. (**d**) Two-dimensional localization error *σ*_*xy*_ measured for a single QD (solid blue) and theoretical precision (see text). Inset shows 200 localizations using *N*=1,500 photons for a single QD, where the localization precision is *σ*_*xy*_=20 nm. Scale bar, 100 μm.

Figure 2 presents a typical measurement of a single QD emitter. A photon correlation measurement, commonly performed in single-particle spectroscopy experiments, is shown in Fig. 2a. Photon antibunching, manifested by a full dip in the autocorrelation function of the photon stream at zero delay (*τ*=0), ensures that indeed the fluorescence source is a single QD emitter. Photoluminescence (PL) rates shown along with *g*^(2)^(0) time traces (Fig. 2b) illustrate that the value of *g*^(2)^(0) remains constant about zero throughout the entire measurement time, whereas the PL fluctuates due to blinking. This comparison highlights the advantage of using the stable photon correlation signal versus using the fluctuating PL intensity signal for estimating the number of emitters in a scene. Single-particle tracking can be performed on the same photon trace to localize the emitter and analyse localization precision. We apply a least squares minimization algorithm to fit the position of an emitter for an *N*=1,500 photons image (Fig. 2c) using a Gaussian point spread function. A standard error for two-dimensional localization 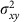
=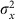
+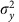 (*σ*_*i*_ is the standard error for localization along axis *i*) is calculated using consecutive localizations of a single QD over 6 s to test the precision of the localization procedure^8^. We compare this precision with a theoretical model^28^ accounting for the pixel size and background counts (Fig. 2d) that follows shot-noise scaling. The localization error of our system departs from this model at high *N*, possibly due to mechanical drift of the sample. An image drift on the scale of a 100 nm in 50 s of measurement was analysed for several scenes of particles, and no individual motion of the particles was resolved (Supplementary Fig. 2).

### Two emitters

**Figure 3:**
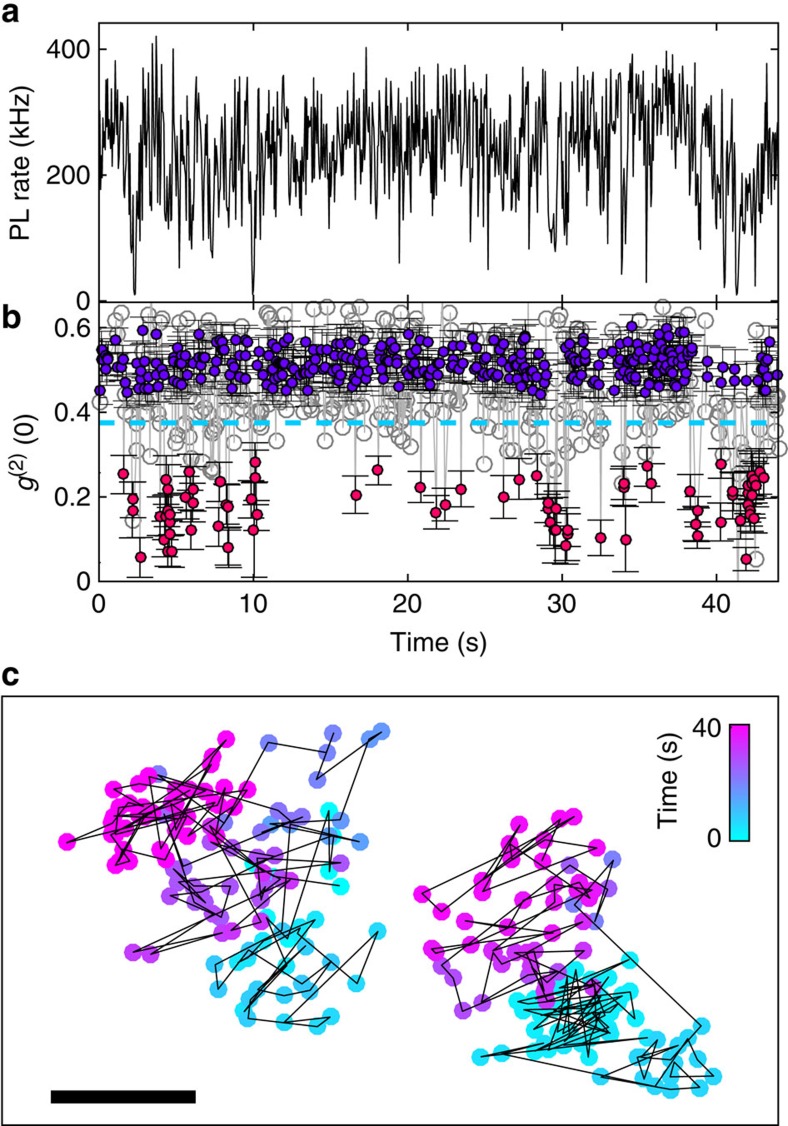
Super-resolution localization and single-particle tracking using quantum correlations of two emitters undergoing drift. (**a**) Photon count and (**b**) quantum correlation for two quantum dots (QDs). Blinking of one QD results in antibunching, *g*^(2)^(0)≈0, shown in red. Instances where more than one emitter is blinked on (blue circles) and where photon statistics is insufficient to count emitters (grey circles) are also shown. Error bars represent ±*σ* statistical error. (**c**) Single-particle tracking (SPT) by repeated localization of two QDs. The separation between the QDs is ∼100 nm. Scale bar, 50 nm.

By using the localization precision together with the extra information provided by photon correlations we demonstrate super-resolved tracking through an analysis algorithm outlined below and detailed in Supplementary Note 2. The PL rates (Fig. 3a) shown alongside the second-order photon correlation function *g*^(2)^(*τ*) (Fig. 3b) are measured and analysed in time bins of 0.1 s. To perform single-emitter localization, we postselect time bins in which only a single emitter was blinked on by thresholding the value of *g*^(2)^(0) below 0.375 (red circles). Instances in which more than one emitter is blinked on, having a higher photon correlation *g*^(2)^(0) value (blue circles), are rejected by the single-emitter algorithm, as well as those with insufficient photon statistics (clear grey circles). Selecting a threshold of *g*^(2)^(0)=0.375 rather than the 0.5 value, inferred from equation (1) for *n*=2 emitters, takes into account some dispersion of PL intensities within the QD ensemble (a detailed derivation of the single-particle criterion can be found in Supplementary Notes 3 and 4).

Localization and tracking of two emitters using our single-emitter localization algorithm for segments composed of *N*=1,500 photons are shown in Fig. 3c. The two emitters separated by ∼100 nm are clearly distinguishable as they move toward the top and left corner of the image. Additional examples of distinguishing two emitters with subwavelength separation are found in Supplementary Fig. 3. The imaging resolution in this case is given by the single-emitter localization precision *σ*_*xy*_, measured to be 20 nm for *N*=1,500 photons (Fig. 2d). In this example, the two QDs are immobilized on the glass substrate while their movement is a result of sample drift, accounting for their correlated motion.

**Figure 4:**
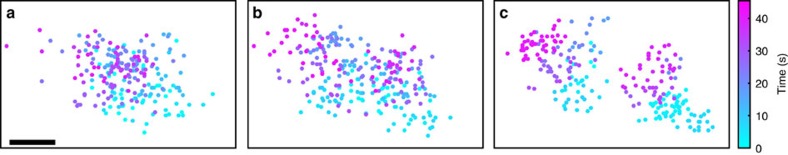
Localization of two QDs with different post-selection criteria. (**a**) Used all photon counts, (**b**) used a photon count rate threshold of the lowest 10% intensity periods and (**c**) used the single-emitter antibunched photon counts (as in Fig. 2c). All localizations use *N*=1,500 photons. For clarity of comparison, (**a**) contains a random selection of 200 data points. Scale bar, 50 nm.

Figure 4 compares postselection based on a quantum-correlation criterion with postselection according to PL intensity for the localization of a pair of blinking QDs. First, localization without any postselection, shown in Fig. 3a, results in scattered points that do not resolve the underlying two-emitter structure. One might expect that postselecting localizations from low brightness periods may reveal single-emitter events without employing photon correlations. However, the localization scatter from the lowest 10% intensity periods, shown in Fig. 4b, does not resolve the two emitters. This is due to a significant number of short PL intermittencies resulting in localized points between the two emitters that obscure the separation of emitter localizations. In contrast, Fig. 4c shows the same data analysed with the single-emitter criterion, clearly resolving the trajectories of the two emitters.

## Discussion

The single-photon fibre bundle camera design allows to image a confocal spot onto an array of a few detectors with a high efficiency. Scaling up this approach to achieve a high coupling efficiency with multiple detectors in a SPAD arrays^29^,^30^ can significantly improve upon their current detection probabilities, limited by low fill factors (typically <10%). Using a large number of fibres to guide light into a SPAD array would enable single-photon sensitive wide-field imaging with a high temporal resolution. In particular, such a design could be used to extend our technique to perform faster super-resolution localization microscopy in widefield by making use of quantum correlations. Furthermore, our current optical configuration combines SPADs with conventional confocal microscopy and could speed-up and enhance the sensitivity of some techniques that image or localize the confocal spot, in particular, confocal super-resolution modalities^3^,^31^,^32^,^33^.

Our technique relies on two requirements for the emitters: first, they must be single-photon emitting, a requirement met by many fluorophores, including organic dyes and certain fluorescent proteins^21^,^34^,^35^,^36^. A second requirement is the detection of a sufficient number of photon correlations events. Common fluorescence microscopy, which uses continuous-wave excitation of organic molecules well below the saturation power of emitters, would typically result in a low number of photon pair detections. In contrast, a pulsed excitation scheme suppresses photobleaching through the triplet state^37^,^38^ and therefore allows the use of pulses with almost unity probability of excitation. In fact, photon correlation measurements with commonly used dye molecule fluorophores were performed under experimental conditions very similar to those used in this work^21^. We emphasize that long-term photostability is not prerequisite from emitters used for super-resolution in our scheme since in particular, 0.1 s were enough to extract the valuable *g*^(2)^ information.

Temporal resolution of super-resolution localization microscopy is limited mainly by the demand of sparse photoswitching^7^,^8^. Namely, to avoid multi-emitter localization events, imaging density should be an order of magnitude lower than one emitter per diffraction-limited spot^39^,^40^. Our approach, integrated into super-resolution localization microscopy, allows to surpass this requirement by precise measurement of the sparsity. By using quantum correlations we measure the number of excited emitters, as follows from equation (1). One can then reject multi-emitter data subsets for single-molecule localizations or even use multi-emitter fitting algorithms^8^,^12^ given the exact number of emitters as extra information. Our results demonstrate localizations of only two emitters; however, we note that our methods would work for more than two emitters as well. In this case, we note that it would become beneficial to make use of emitters that have faster blinking statistics than the ones used here to facilitate the occurrence of single-emitter events and localizations of scenes of three emitters or more at viable performance.

Other techniques that achieve super-resolved images using photon and image correlations show an improvement of the resolution as the square root of the highest order of calculated correlation^6^,^19^,^41^. Practically, signal-to-noise and low contrast in high-order correlation pose a limiting factor for such an improvement^6^,^19^. In super-resolution optical fluctuation imaging^6^, for example, imaging of two emitters with a fivefold resolution improvement was acquired in several minutes, whereas we demonstrate a 10-fold enhancement in resolution with a temporal resolution of seconds for a small field of view.

To summarize, we presented a method that applies quantum photon correlations to accurately localize emitters within a diffraction-limited spot. To acquire an image together with photon correlations, we utilized a few-pixel confocal camera using a fibre bundle combined with SPADs. Replacing a standard detector of a confocal microscope with the fibre bundle system described above can potentially speed up super-resolved localization microscopy by alleviating the frame sparsity constraint.

## Methods

### Microscope setup

An optical microscope (Zeiss Axiovert 135) is used to image fluorescent samples of QDs. A two-axis piezo stage (P-542.2SL, Physik Instrumente) is used to position the sample. For illumination, a 473 nm pulsed picosecond laser diode (Edinburgh Instruments) is used, coupled to a single-mode fibre. The repetition rate of this laser is set to 20 MHz. A 1.4 numerical aperture objective lens (Plan Apo Vc 100 × , Nikon) is used to tightly focus the illuminating laser. The fluorescence is collected by the same objective lens and filtered by dichroic mirrors and filters (FF509-FDi01, SP01-785RS, BLP01-532R, Semrock). A Galilean beam expander (BE05-10-A, Thorlabs) is placed following the relay lens to magnify the imaged fluorescence spot on to a fibre bundle (A.R.T. Photonics GmbH, Germany). This fibre bundle consists of multimode 100/110 μm core/clad fibres, fused on one side and fan-out to individual multimode fibres on the other side, and is used to guide photon from an imaged spot to 15 fibre coupled single-photon avalanche photodiodes (SPCM-AQ4C, Perkin-Elemer). For a detailed characterization of the fibre bundle setup see Supplementary Note 4. The overall detection efficiency of our setup is 12%, and further details about the efficiency are found in the Supplementary Note 1 and Supplementary Table 1.

### Data acquisition and analysis

A time-correlated single-photon counting board is used for data acquisition in absolute timing mode (DPC-230, Becker & Hickl GmbH). An excitation pulse trigger is synchronized and recorded at every 40th pulse (0.5 MHz). The correlation analysis and localization algorithms (Supplementary Software 1) were implemented in a MATLAB script, postprocessing the acquired data. Further details about the algorithms are found in Supplementary Fig. 4 and Supplementary Note 5.

### QDs and sample preparation

Samples of CdSe/CdS/ZnS colloidal QDs^19^ were prepared by spin coating a low concentration solution mixed with poly(methyl methacrylate) on a microscope coverslips. Fluorescence from the these QDs peaks at 610 nm, with a lifetime of 26 ns.

### Data availability

The raw data that support the findings of this study are available in figshare repository with the identifier doi:10.6084/m9.figshare.4588723.v1 (ref. 42).

## Additional information

**How to cite this article:** Israel, Y. *et al*. Quantum correlation enhanced super-resolution localization microscopy enabled by a fibre bundle camera. *Nat. Commun.* **8,** 14786 doi: 10.1038/ncomms14786 (2017).

**Publisher's note**: Springer Nature remains neutral with regard to jurisdictional claims in published maps and institutional affiliations.

## Supplementary Material

Supplementary Information Supplementary Figures, Supplementary Table and Supplementary Notes.

Supplementary Software 1 This software package contains the algorithms for calculating g^(2), thresholding single-emitter events and the localization algorithm used in the manuscript.

## References

[b1] Abbe E. Beiträge zur theorie des mikroskops und der mikroskopischen wahrnehmung. Arch. Mikrosk. Anat. 9, 413–418 (1873).

[b2] Rayleigh L. On the theory of optical images, with special reference to the microscope. Philos. Mag. 42, 167–195 (1896).

[b3] Klar T. A., Jakobs S., Dyba M., Egner A. & Hell S. W. Fluorescence microscopy with diffraction resolution barrier broken by stimulated emission. Proc. Natl Acad. Sci. USA 97, 8206–8210 (2000). 10899992 10.1073/pnas.97.15.8206 PMC26924

[b4] Rust M. J., Bates M. & Zhuang X. Sub-diffraction-limit imaging by stochastic optical reconstruction microscopy (STORM). Nat. Methods 3, 793–796 (2006). 16896339 10.1038/nmeth929 PMC2700296

[b5] Betzig E. . Imaging intracellular fluorescent proteins at nanometer resolution. Science 313, 1642–1645 (2006). 16902090 10.1126/science.1127344

[b6] Dertinger T., Colyer R., Iyer G., Weiss S. & Enderlein J. Fast, background-free, 3d super-resolution optical fluctuation imaging (SOFI). Proc. Natl Acad. Sci. USA 106, 22287–22292 (2009). 20018714 10.1073/pnas.0907866106 PMC2799731

[b7] Weisenburger S. & Sandoghdar V. Light microscopy: an ongoing contemporary revolution. Contemp. Phys. 56, 123–143 (2015).

[b8] Deschout H. . Precisely and accurately localizing single emitters in fluorescence microscopy. Nat. Methods 11, 253–266 (2014). 24577276 10.1038/nmeth.2843

[b9] Small A. R. Theoretical limits on errors and acquisition rates in localizing switchable fluorophores. Biophys. J. 96, L16–L18 (2009). 19167282 10.1016/j.bpj.2008.11.001 PMC2716449

[b10] Huang F., Schwartz S. L., Byars J. M. & Lidke K. A. Simultaneous multiple-emitter fitting for single molecule super-resolution imaging. Biomed. Opt. Express 2, 1377–1393 (2011). 21559149 10.1364/BOE.2.001377 PMC3087594

[b11] Zhu L., Zhang W., Elnatan D. & Huang B. Faster STORM using compressed sensing. Nat. Methods 9, 721–723 (2012). 22522657 10.1038/nmeth.1978 PMC3477591

[b12] Huang F. . Video-rate nanoscopy using scmos camera-specific single-molecule localization algorithms. Nat. Methods 10, 653–658 (2013). 23708387 10.1038/nmeth.2488 PMC3696415

[b13] Barsic A. & Piestun R. Super-resolution of dense nanoscale emitters beyond the diffraction limit using spatial and temporal information. Appl. Phys. Lett. 102, 231103 (2013).

[b14] Wang Y., Quan T., Zeng S. & Huang Z.-L. PALMER: a method capable of parallel localization of multiple emitters for high-density localization microscopy. Opt. Express 20, 16039–16049 (2012). 22772294 10.1364/OE.20.016039

[b15] Small A. & Stahlheber S. Fluorophore localization algorithms for super-resolution microscopy. Nat. Methods 11, 267–279 (2014). 24577277 10.1038/nmeth.2844

[b16] Schwartz O. & Oron D. Improved resolution in fluorescence microscopy using quantum correlations. Phys. Rev. A 85, 033812 (2012).

[b17] Nair R. & Tsang M. Far-field superresolution of thermal electromagnetic sources at the quantum limit. Phys. Rev. Lett. 117, 190801 (2016). 27858425 10.1103/PhysRevLett.117.190801

[b18] Lupo C. & Pirandola S. Ultimate precision bound of quantum and subwavelength imaging. Phys. Rev. Lett. 117, 190802 (2016). 27858426 10.1103/PhysRevLett.117.190802

[b19] Schwartz O. . Superresolution microscopy with quantum emitters. Nano Lett. 13, 5832–5836 (2013). 24195698 10.1021/nl402552m

[b20] Cui J.-M., Sun F.-W., Chen X.-D., Gong Z.-J. & Guo G.-C. Quantum statistical imaging of particles without restriction of the diffraction limit. Phys. Rev. Lett. 110, 153901 (2013). 25167270 10.1103/PhysRevLett.110.153901

[b21] Grußmayer K. S., Kurz A. & Herten D.-P. Single-molecule studies on the label number distribution of fluorescent markers. Chemphyschem 15, 734–742 (2014). 24677641 10.1002/cphc.201300840

[b22] Ta H. . Mapping molecules in scanning far-field fluorescence nanoscopy. Nat. Commun. 6, 7977 (2015). 26269133 10.1038/ncomms8977 PMC4557268

[b23] Chao J., Ram S., Ward E. S. & Ober R. J. Ultrahigh accuracy imaging modality for super-localization microscopy. Nat. Methods 10, 335–338 (2013). 23455923 10.1038/nmeth.2396 PMC3833351

[b24] Krishnaswami V., Van Noorden C. J., Manders E. M. & Hoebe R. A. Towards digital photon counting cameras for single-molecule optical nanoscopy. Opt. Nanoscopy 3, 1 (2014).

[b25] Pavia J. M., Wolf M. & Charbon E. Measurement and modeling of microlenses fabricated on single-photon avalanche diode arrays for fill factor recovery. Opt. Express 22, 4202–4213 (2014). 24663744 10.1364/OE.22.004202

[b26] Lounis B. & Orrit M. Single-photon sources. Rep. Prog. Phys. 68, 1129–1179 (2005).

[b27] Gerry C. C. & Knight P. L. Introductory Quantum Optics Cambridge University Press (2005).

[b28] Mortensen K. I., Churchman L. S., Spudich J. A. & Flyvbjerg H. Optimized localization analysis for single-molecule tracking and super-resolution microscopy. Nat. Methods 7, 377–381 (2010). 20364147 10.1038/nmeth.1447 PMC3127582

[b29] Cova S. D. & Ghioni M. Single-photon counting detectors. Photonics J. IEEE 3, 274–277 (2011).

[b30] Charbon E. & Fishburn M. W. in Single-Photon Imaging (eds Seitz, P. & Theuwissen, A. JP) 123–157Springer (2011).

[b31] Müller C. B. & Enderlein J. Image scanning microscopy. Phys. Rev. Lett. 104, 198101 (2010). 20867000 10.1103/PhysRevLett.104.198101

[b32] Rosen S., Sirat G. Y., Ilan H. & Agranat A. J. A sub wavelength localization scheme in optical imaging using conical diffraction. Opt. Express 21, 10133–10138 (2013). 23609718 10.1364/OE.21.010133

[b33] Huff J. The Airyscan detector from ZEISS: confocal imaging with improved signal-to-noise ratio and super-resolution. Nat. Methods 12, 12 (2015).

[b34] Lounis B. & Moerner W. E. Single photons on demand from a single molecule at room temperature. Nature 407, 491–493 (2000). 11028995 10.1038/35035032

[b35] Fernández-Suárez M. & Ting A. Y. Fluorescent probes for super-resolution imaging in living cells. Nat. Rev. Mol. Cell Biol. 9, 929–943 (2008). 19002208 10.1038/nrm2531

[b36] Han J. J., Kiss C., Bradbury A. R. & Werner J. H. Time-resolved, confocal single-molecule tracking of individual organic dyes and fluorescent proteins in three dimensions. ACS Nano 6, 8922–8932 (2012). 22957739 10.1021/nn302912j

[b37] Donnert G., Eggeling C. & Hell S. W. Major signal increase in fluorescence microscopy through dark-state relaxation. Nat. Methods 4, 81–86 (2007). 17179937 10.1038/nmeth986

[b38] Jacques V. . Enhancing single-molecule photostability by optical feedback from quantum jump detection. Appl. Phys. Lett. 93, 203307 (2008).

[b39] Min J. . FALCON: fast and unbiased reconstruction of high-density super-resolution microscopy data. Sci. Rep. 4, 4577 (2014). 24694686 10.1038/srep04577 PMC3974135

[b40] Siewert S. . Sparse deconvolution of high-density super-resolution images. Sci. Rep. 6, 21413 (2016). 26912448 10.1038/srep21413 PMC4766479

[b41] Gatto Monticone D. . Beating the Abbe diffraction limit in confocal microscopy via nonclassical photon statistics. Phys. Rev. Lett. 113, 143602 (2014). 25325642 10.1103/PhysRevLett.113.143602

[b42] Tenne R., Israel Y., Oron D. & Silberberg Y. ExampleData2.zip (2017) URL https://figshare.com/articles/ExampleData2_zip/4588723. 10.1038/ncomms14786 PMC5355801 28287167

