# Supplementary material for: Quantum correlation enhanced super-resolution localization microscopy enabled by a fibre bundle camera
Source: Nat Commun. 2017 Mar 13;8:14786. doi: 10.1038/ncomms14786 (PMC5355801; doi:10.1038/ncomms14786)
Supplement: Supplementary Information — Supplementary Figures, Supplementary Table and Supplementary Notes. [file ncomms14786-s1.pdf]

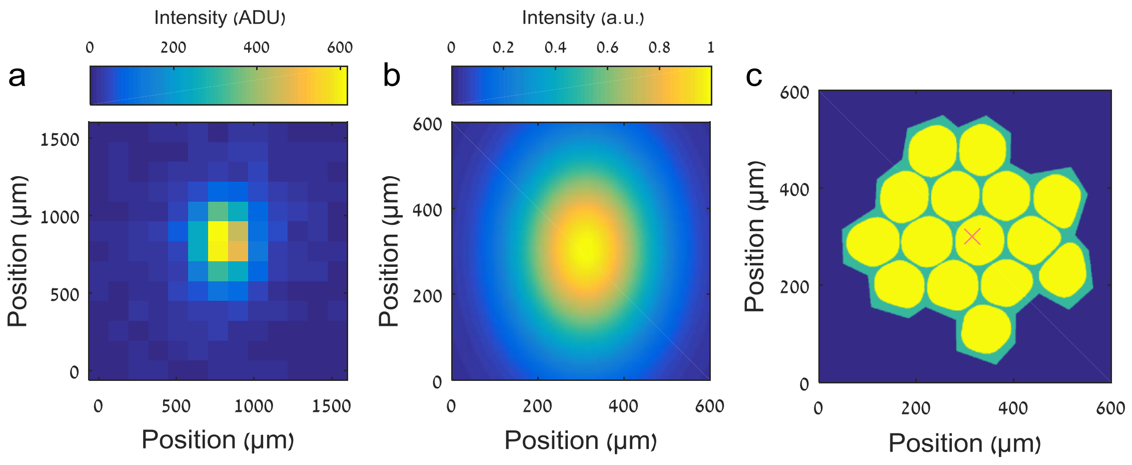

Supplementary Figure 1: Estimation of loss in the fiber bundle. In order to extract the coupling efficiency of light into the fiber bundle we compare the number of detected photons in the CCD and fiber bundle. (a) An image of a 20nm fluorescent bead taken with a CCD camera with a 0.1s exposure. The intensity is given in analog to digital units (ADU) which was converted into the number of detected photons according to the manufacturer (Rollera Thunder, QImaging) specifications. (b) A Gaussian fit of the image shown in (a). (c) A segmented image of the fiber bundle used in our setup. Fibers are shown in yellow whereas a red cross marks the center of the PSF, shown in (b), on the fiber bundle image. An estimate of the photon rate impinging on the fiber bundle is calculated by integrating the Gaussian PSF fit function on the active area encircling the fibers shown in light green in (c). Further details about the estimation of loss can be found in Supplementary Note 1.

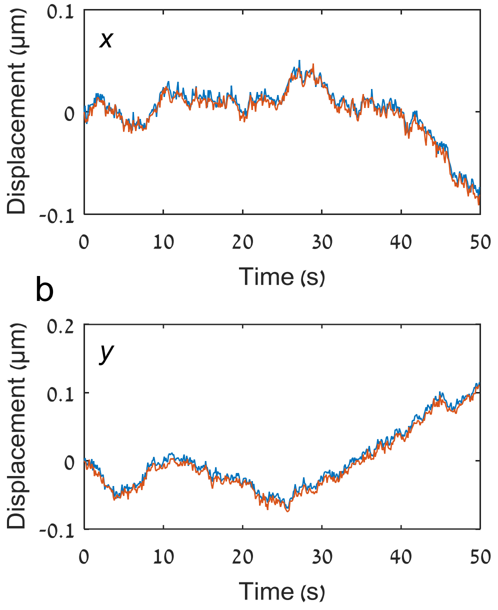

Supplementary Figure 2: Measurement of sample drift. A fluorescent bead sample (1μm diameter, 560nm emission, Molecular Probes) was prepared by drop casting a diluted solution onto a glass coverslip to create a sparse scene of bright spots. Sequential frames, taken 120ms apart (exposure time of 100ms), were fitted with a 2D-Gaussian to localize two of the beads in each frame. Displacements in the x (a) and y (b) directions of each bead from its position in the first frame are plotted versus time over a period of 50s. A drift in a scale of a 100nm in 50 seconds of measurement is clearly visible in these trajectories whereas no individual motion of the particles can be resolved. This scale of motion measured for several different scenes is similar to the one measured with the photon correlation based localization algorithm presented in Figure 3.

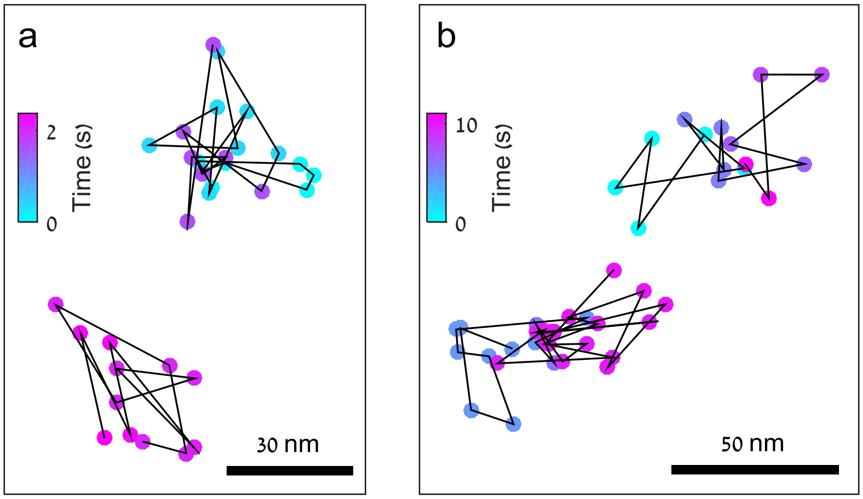

Supplementary Figure 3: Further examples of super-resolved localizations of two QDs. QDs are separated by (a) 64nm, and (b) 78nm. Localizations use $N = 1500$ photons per localization.

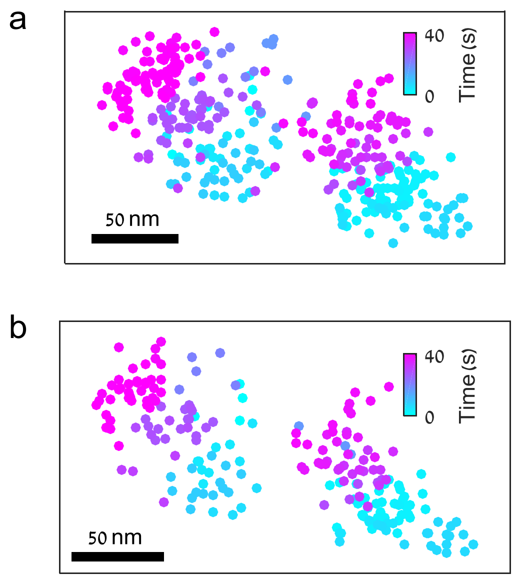

Supplementary Figure 4: Comparison between two particle tracking without (a) and with (b) employing the rejection criteria described in Supplementary Note 2. Marker color denotes time from the beginning of the measurement.

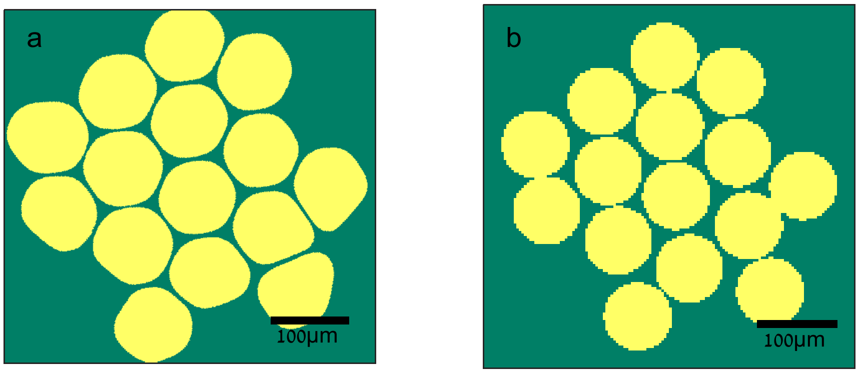

Supplementary Figure 5: A comparison between a segmented image of the fiber bundle (a) input plane and a spherical fiber approximation of that image (b) composed for the purpose of computational speed-up. The segmented image was compiled by thresholding an array of images in which light was input into one of the fibers. Further details regarding the characterization of the fiber bundle geometry and variations in single photon avalanche detector (SPAD) detection efficiencies can be found in Supplementary Note 5.

Loss component	Efficiency
Objective transmission (Plan Apo Vc 100X, Nikon)<br>collection<br>transmission	 50%<br>86%
Dielectric filters and dichroic mirror (FF509-FDi01, SP01-785RS, BLP01-532R, Semrock)	95%
Additional lenses and beam expander	95%
Beam splitter	88%
Fiber bundle (confocal efficiency, of that 82% is the collection efficiency)	60%
SPAD (SPCM- AQ4C, Perkin-Elemer)	60%
**Overall**	**12%**

Supplementary Table 1: Loss contribution specified for the different optical components present in our setup. First, the collection efficiency of fluorescence by a high numerical aperture (NA) objective is estimated to be about 50% (NA 1.4, oil immersion), where its transmission at the fluorescence peak (610nm) is about 86% (product specifications). Spectral filtering using a dichroic mirror and two dielectric filters account for an additional 5% loss. Other optical elements in the setup, including the tube lens, relay lens and beam expander, are coated with an anti-reflective coating and introduce additional loss estimated at about 5%. A 90/10 beam splitter is placed in front of the fiber bundle directing about 10% of the light towards a CCD camera. Its transmission was measured to be 88% for unpolarized light. As detailed in Supplementary Note 1 the collection efficiency of the fiber bundle from a confocal spot is 60%, limited mainly by our fiber bundle collection area, collecting about 82% of the light impinging that area. An imperfect detector's quantum efficiency contributes to the loss of 40% of the photons that reach the SPADs. These factors of loss amount to an overall efficiency (ratio of detected photons to photons emitted from a diffraction limited spot) of 12% in our setup.

Supplementary Note 1: Measurement of fiber bundle collection efficiency

We present in the supplementary information an analysis of the loss of fluorescence in our microscopy setup separated into two parts. Supplementary Table 1 mostly accounts for loss in typical optical components used in confocal microscopy. In this note we address the loss in the detection system we introduced, namely, a fiber bundle to guide light from a confocal spot to several single-photon avalanche detectors (SPADs). While most of the optical attenuation occurs due to components in the first part, we highlight here the characterization of loss in coupling into the fiber bundle and propagation within the fibers themselves, a unique property of our optical setup, which allows to efficient guiding of photons to SPADs.

Two major factors account for loss in the fiber bundle: light lost in the space between fibers and light from the confocal spot lost outside of the fiber bundle collection area. While the second loss mechanism is practically important considering the small number of detectors in our setup, it can be neglected for a large bundle area. On the other hand, the first factor is present in any fiber bundle imaging scheme.

In the following we define and estimate two coupling efficiencies: the first corresponds to the upper limit of coupling through a fiber bundle accounting only for the first loss factor and is termed here the coupling efficiency $\alpha_{\mathrm{coupling}}$. We term the second estimate the confocal efficiency $\alpha_{\mathrm{confocal}}$, which considers all light lost from the imaging of a point-like fluorophore, accounting for both of the aforementioned loss factors and is particular for the setup presented in this work. Note that both efficiencies do not include loss in any element other than the fiber bundle itself in order to isolate its effect on the detection probability.

In order to estimate the coupling efficiency of light to the fiber in the fiber bundle we prepared a sample of 20nm fluorescent beads (Thermo-Fisher F8786) drop casted sparsely onto a cover slip. We image a single bead and split its fluorescence using a 90/10 beam-splitter (following the beam expander), where about 10% of the light is directed to a reference CCD camera (Rolera Thunder, QImaging) and about 90% of the light is transmitted onto the fiber bundle setup. Taking into account the quantum efficiencies of the CCD and SPADs as specified by the manufacturers we can extract the fiber bundle coupling efficiency from the ratio of detected number of photons in these two detectors over a similar acquisition time.

While estimating the number of photons detected in the SPADs (I_{SPAD}) in a time period T is a straightforward task, some analysis is needed in order to extract a comparable photon number from the camera measurement. First we subtract from a CCD frame an average of 20 background frames recorded at dark conditions (all frames with exposure time T).

We define here the fiber bundle coupling efficiency as the probability of a photon that impinges upon an area encompassing the active fibers to reach the SPADs. Supplementary Figure 1c shows the region around the fiber which we term the active area roughly circling the outer fibers. To estimate the coupling efficiency we evaluate the number of photon measured by the CCD in the region equivalent to the active area of the fiber bundle ($I_{\mathrm{CCD-ROI}}$). This evaluation is performed

by fitting the CCD frame to a 2D Gaussian (Supplementary Figure 1b) function and integrating it over the active area. . The coupling efficiency is then given by:

$$\alpha_{\text{coupling}} = \frac{I_{\text{SPAD}} \times P_{\text{SPAD}}}{I_{\text{CCD-ROI}} \times P_{\text{CCD}}} \tag{1}$$

Here, P_{SPAD} (P_{CCD}) is the probability that a photon impinging upon the beam splitter will be detected by the SPADs (CCD), without considering loss in the fiber bundle:

$$\begin{aligned} P_{\text{SPAD}} &= T_{\text{BS}} \times QE_{\text{SPAD}} = 0.53, \\ P_{\text{CCD}} &= R_{\text{BS}} \times QE_{\text{CCD}} = 0.10, \end{aligned} \tag{2}$$

where T_{BS} (R_{BS}) is the transmission (reflection) coefficient for the 90/10 beam splitter as given by the manufacturer's specifications and QE_{SPAD} (QE_{CCD}) is the quantum efficiency of the SPADs (CCD).

Applying the experimental results to Supplementary Equations 1 and 2 yields a coupling efficiency of 82%. Note that the geometrical fill factor, that is. ratio between the total fiber area and the selected encompassing region of interest, is 75%. The relatively good agreement between these two numbers indicates that the main cause of loss in the fiber bundle is the inactive area separating the fibers while reflection at the entrance plane and exit plane and attenuation within the fibers are negligible. The coupling of light into SPADs in this design is significantly better than that of SPAD arrays whose record fill factor is about 10%.

We define here the confocal efficiency as the probability of a photon emitted from a point-like source and reaching the fiber bundle to be guided into the SPADs. To estimate it we first subtract the background fluorescence (who is broader than the active area) from the bead measurements in the CCD and the SPAD, taking only the number of photons emitted from the fluorescent bead. We do so by subtracting an estimate of the background fluorescence extracted from the aforementioned Gaussian fit (B_{SPAD}) from the number of photons detected by the SPADs. The confocal efficiency can then be extracted from:

$$\alpha_{\text{confocal}} = \frac{(I_{\text{SPAD}} - B_{\text{SPAD}}) \times P_{\text{SPAD}}}{I_{\text{Guassian}} \times P_{\text{CCD}}}, \tag{3}$$

where I_{Guassian} is the integration of the number of detected photons in the Gaussian component in the fit of the CCD frame.

Applying the experimental results to Supplementary Equation 3 yields a confocal efficiency of 60%. This number is used in Supplementary Table 1 to estimate the overall loss of fluorescence in our experiments. It includes both the loss of light collection due to boundary areas between fibers as well as the fringes of the point-spread function which are imaged outside of the active area. While this efficiency is essential for the setup presented in this work, it is dependent upon the magnification and could be enlarged by decreasing it or by using a larger array of fiber coupled SPADs.

Supplementary Note 2: The localization algorithm

Below is a detailed description of the localization algorithm applied to the raw data collected by our experimental setup. The algorithm was implemented with a custom MATLAB script post-processing data acquired throughout the acquisition time. The same algorithm was used to analyze all data traces without any parameter adjustments.

We initially parse the data of a photon trace in all detectors into time bins of $T = 0.1$ s duration. For each such time bin we calculate the function $G^{(2)}(m)$ by counting the number of detection pairs with time delays in the range of $m \times \Delta t \leq \tau < (m+1) \times \Delta t$. Here, $m = 0,1, \dots ,50$ and Δt is the time between subsequent excitation pulses. We then apply Supplementary Equation 8 to evaluate the antibunching value, $g^{(2)}(0)$.

A time bin with a value of $g^{(2)}(0)$ below the threshold of 0.375 with a statistical significance of 95%, that is more than 1.96 standard deviations below the threshold value (see Supplementary Notes 3 and 4), indicates that a single particle is present in the observed scene.

Each of the time windows is parsed into blocks of $N = 1500$ consecutive photon detections. An image is constructed by dividing these detections according to the fiber through which they passed and subtracting the dark current estimate for each detector, as shown in Figure 2b. In order to correct for image distortion due to the non-uniform detection efficiency in SPADs, we divide the number of detections in each fiber by its calibrated relative efficiency (see Supplementary Note 5).

The corrected image is fitted using a 2D Gaussian model for the point-spread-function (PSF) and a least-mean-squares fitting method. This fit is performed using 7 parameters: x and y of center positions, σ_x and σ_y Gaussian PSF width, θ orientation angle, an amplitude and background. The use of the PSF widths and orientation angle as fitting parameter accounts for variations in focus conditions between different measurements. Indeed, these parameters' values remain stable within each specific measurement implying that the number of fitting parameters used could be decreased.

Two rejection criteria are employed to reject some artifacts of the localization blocks. The first criterion rejects outlying localizations in which the fit did not succeed concluding with PSF width values (σ_x or σ_y) outside an expected range of 100-180nm, as characterized from measurements of single QDs. This criterion rejects ~5% of the localization blocks, usually those with low signal-to-background ratio (for example, short on-blinking event of a single QD), and regardless of whether one or two close particles are present in the measurement volume.

The second criterion filters out a case of short events of blinking within a single time window. In these cases, one emitter was switched on for most of the period while the other was on for a short period of time. These blocks therefore contain photons contributed from both emitters while their $g^{(2)}(0)$ is still below the threshold. Such a block would result in a localized point that is in between the two emitters hindering the resolution of close particles.

In order to address this problem by locating such peculiar blocks we define an effective distance between two images as follows:

$$d(i,j)^2 = \sum_k \frac{\left(S_i(k) - S_j(k)\right)^2}{\delta S_i(k)^2 + \delta S_j(k)^2} = \sum_k \frac{\left(S_i(k) - S_j(k)\right)^2}{S_i(k) + S_j(k)} \quad (4)$$

Here, $S_i(k)$ is the number of detections measured in the i-th block in the k-th detector. The last equality assumes a shot-noise variation in the number of detected photons. Simply put, $d(i,j)$ is the number of standard deviations in which the i-th image differs from the j-th image. We reject a block if its distance from one of the other images in the same time bin exceeds a value of 1.7. This criterion rarely rejects any block in the case of a single emitter measurement while in the case of two emitters it is useful to reject localization centered between the locations of the two emitters. Supplementary Figure 4 shows a comparison of the same data shown in Figure 3b in the main text, with and without the distance rejection criterion. While the distribution of localizations is wider without applying the above criteria, the two tracked objects are still well separated. We also note that in the preparation of figure 4 of the manuscript the same rejection criteria were used in all panels.

Supplementary Note 3: Derivation of single particle $g^{(2)}(0)$ threshold value using the case of two particles

Photon antibunching is a signature of photoluminescence (PL) of a single emitter, characterized by a nearly zero probability of detecting two photons simultaneously. Under a short pulse excitation a single QD emits at most a single photon per period of $\Delta t = \frac{1}{f_{\text{pul}}}$, where f_{pul} is the laser pulse repetition rate. Therefore simultaneous detection pairs exclusively result from multiple emitters in the 'on' state at the same time within the measured volume. To distinguish between the cases of single and multiple emitting QDs we analyze the limiting case of two emitting QDs and calculate the expected photon correlation under pulsed excitation.

The total photon detection rate for a single emitter is $I = p \times f_{\text{pul}}$. where p is the probability for detection per pulse ($p \ll 1$). For two emitters, this rate is combined from the contributions of $I_1 = \alpha \times p \times f_{\text{pul}}$ and $I_2 = (1-\alpha) \times p \times f_{\text{pul}}$, that is $I = I_1 + I_2$, where α is the fraction of photon rate of the first emitter from the total (assuming the first emitter is the brighter one, $0.5 \leq \alpha \leq 1$).

Assuming the photon emission of the first emitter is uncorrelated with that of the second, the number of photon pairs detected with a delay of less than Δt (i.e. from the same excitation pulse) within a period of duration T:

$$G^{(2)}(m=0) = 2(\alpha \cdot p) \times \big((1-\alpha) \times p\big) \times f_{\text{pul}} \cdot T = 2\alpha \times (1-\alpha) \times p^2 \times f_{\text{pul}} \times T \tag{5}$$

Here $G^{(2)}(m)$ is the number of photon pairs over delays of $m \times \Delta t \leq \tau < (m+1) \times \Delta t$. Considering there is no correlation in the probabilities of emission of a QD for delays greater than Δt, that is no memory after the exciton recombination, the number of photon pairs delayed by $m \geq 1$ pulses is:

$$G^{(2)}(m \geq 1) = p^2 \times f_{\text{pul}} \times T \tag{6}$$

Taking the ratio between pair detections at zero and non-zero ($m < 50$) pulse delays we get the second order quantum correlation:

$$g^{(2)}_{n=2}(\tau = 0) = \frac{G^{(2)}(m=0)}{G^{(2)}(\tau = m)} = 2\alpha \times (1-\alpha) \tag{7}$$

For equally bright emitters ($\alpha = 0.5$) we obtain a maximal value of $g^{(2)}_{n=2}(\tau = 0)$, concurring with Eq. 1 (main text) for $n = 2$:

$$g^{(2)}_{n=2}(\tau = 0) = \frac{1}{2} \tag{8}$$

Since an ensemble of QDs has a non-uniform quantum yield and absorption cross-section, leading to inhomogeneity in the time-averaged photon emission rate between different single QDs, the value of $g^{(2)}_{n=2}(0)$ may be lower than 0.5. Taking into account reasonable values for this inhomogeneity, we set the threshold for a single QD at a value of $g^{(2)}_{n=2}(0) = 0.375$ for the case of $\alpha = 0.75$. We therefore set the single QD criterion to accept only events in which at least 75% of the PL photons are emitted from a single QD.

Supplementary Note 4: Statistical analysis of the single particle criterion

The measured value of $g^{(2)}(0)$ is analyzed by integration of detected counts over a time bin. In order to account for the measurement uncertainties in the implementation of the single particle criterion, we treat in the following the statistical error in the estimate of $g^{(2)}(0)$. The criterion for single particles is defined by the measured value of $g^{(2)}(0)$ being below the $g^{(2)}_{n=2}(0) = 0.375$ threshold with over 95% confidence level (CL). Finally, the time bin duration (T) is optimized so that a typical single emitter passes the above criterion (for the QDs we used the optimal time bin duration is T = 100ms).

Additionally, we account for false detection of correlations resulting from detector dark current (detailed discussion of the origin of this background is given below). We estimate the value of $g^{(2)}(0)$ in the following manner:

$$g^{(2)}(0) = \frac{G^{(2)}(0) - B}{\langle G^{(2)} \rangle - B} \tag{9}$$

Here, B is the estimated detection pair count per time bin, arising from photon-dark current and dark current-dark current correlation. $\langle G^{(2)} \rangle$ stands for the mean correlation count, calculated by averaging the measured $G^{(2)}(m)$ function over the range $4 \le m \le 50$. This range of delays was chosen so as not to include short delays in which residual antibunching may occur as well as long delays in which blinking may affect the correlation value.

The root-mean-squared-error (RMSE) in $g^{(2)}(0)$, referred to as the standard error hereafter, follows from the error propagation formula:

$$\sigma = \mathrm{RMSE}[g^{(2)}(0)] = \sqrt{\left[\frac{\partial g^{(2)}(0)}{\partial B}\delta B\right]^2 + \left[\frac{\partial g^{(2)}(0)}{\partial \langle G^{(2)} \rangle}\delta \langle G^{(2)} \rangle\right]^2 + \left[\frac{\partial g^{(2)}(0)}{\partial G^{(2)}(0)}\delta G^{(2)}(0)\right]^2} \tag{10}$$

Below we describe the estimators $G^{(2)}(0)$, $\langle G^{(2)} \rangle$, B and their error estimates separately.

We begin by addressing the first term in Supplementary Equation 9: an error due to variance in detection pairs which include at least one dark current count. For the purpose of the following discussion we disregard dark current–dark current correlations, which are negligible in our measurements, and treat here explicitly background arising from photon-dark current pairs.

B then follows:

$$B = \sum_{i \neq j} \frac{S_i}{N_{\mathrm{pul}}} \times \frac{dc_j}{N_{\mathrm{pul}}} f_{\mathrm{pul}} \times T \tag{11}$$

Here, S_i (dc_i) is the estimate of photon (dark current) detection count in the ith detector over a time bin of duration T and N_{pul} is the number of pulses within that same time window.

Assuming shot-noise in the estimate of both S_i and dc_i and taking into account signal to dark current ratio of 10 results in the following estimate:

$$\delta B^2 = \left(\frac{S \times dc}{N_{pul}}\right)^2 \times \left[\frac{1}{S} + \frac{1}{dc}\right] \tag{12}$$

Where $S(dc)$ is the photon (dark current) detection estimate summed over all detectors.

The second term in Supplementary Equation 9: an error due to errors in the estimate of $\langle G^{(2)}\rangle$. This value is estimated by averaging measurements of $G^{(2)}(m)$ over a range of $4 \leq m \leq 50$. Assuming a Poissonian distribution for the photon correlation counts $G^{(2)}(m)$, the error in $\langle G^{(2)}\rangle$ is

$$\delta\langle G^{(2)}\rangle = \sqrt{\frac{\langle G^{(2)}\rangle}{M}} \tag{13}$$

where M is the number of delays in the averaging range.

The last term in Supplementary Equation 9 results from variations in the detected number of zero delay detection pairs. Assuming a Poissonian distribution for the photon correlation count in zero delay $G^{(2)}(0)$, its error is

$$\delta G^{(2)}(0) = \sqrt{G^{(2)}(0)} \tag{14}$$

Due to shot-noise in the value of $G^{(2)}(0)$, some of the time bins may have a low or even zero count. In these instances the value of $\delta G^{(2)}(0)$ is underestimated, therefore we use a floored value for $G^{(2)}(0)$ of $0.1 \times \langle G^{(2)}\rangle + B$. The value 0.1 is used as a mean value of $g^{(2)}(0)$, based on measurement of a few tens of single CdSe/CdS/ZnS QDs used in this work.

$$\delta G^{(2)}(0) = \max\left\{\sqrt{G^{(2)}(0)}\ , 0.1 \times \langle G^{(2)}\rangle + B\right\} \tag{15}$$

Summing up the contributions of the three terms in Supplementary Equation 9, that is inserting the terms in Supplementary Equations 11,12 and 14 into Supplementary Equation 9, we obtain the full expression for the estimate of the standard error in $g^{(2)}(0)$ in each time bin:

$$\sigma = \sqrt{\left[\frac{G^{(2)}(0) - \langle G^{(2)}\rangle}{(\langle G^{(2)}\rangle - B)^2} \times \delta B\right]^2 + \left[\frac{G^{(2)}(0) - B}{(\langle G^{(2)}\rangle - B)^2} \times \frac{\langle G^{(2)}\rangle}{\sqrt{M}}\right]^2 + \left[\frac{\delta G^{(2)}(0)}{\langle G^{(2)}\rangle - B}\right]^2} \tag{16}$$

Supplementary Equation 15 is implemented in the localization algorithm in order to estimate the error in each measurement time bin and decide whether $g^{(2)}(0)$ is below the threshold level with a 95% confidence level.

Using typical experimental values for a time bin in which only a single QD is present, we estimate $\sigma \sim 0.05$ for a time window of $T = 0.1$ seconds, a photon rate of 10^5 Hz, a dark current rate of 7×10^3 Hz, $g^{(2)}(0) = 0.1$ and a pulse repetition rate of $f_{\text{pul}} = 20$ MHz.

Supplementary Note 5: Collection setup characterization

Since our effective camera is not made out of equal area square pixels as in common cameras, we characterize our fiber bundle and the detection efficiency of our detectors. The objectives of this note are to describe the physical characteristics and discuss the measurement of variations in the detection efficiency within the array. This characterization is implemented in the localization algorithm described in Supplementary Note 2 and is crucial for unbiased localization of emitters.

Supplementary Figure 5a shows an image depicting the fiber bundle input plane obtained by thresholding microscope images of the bundle input plane while illuminating the other end of each of the fibers in the bundle. One can observe that the fibers have an approximately circular profile and are arranged in a slightly distorted honey-comb lattice. Fiber areas are rather uniform with less than 10% difference between largest and smallest and a mean value of $\sim 7900 \mu m^2$, the area of a $100 \mu m$ diameter disk.

In order to reduce the computation complexity of the fitting algorithm we approximate the actual fiber array input plane with circular $100 \mu m$ diameter fibers in the localization algorithms used throughout the paper. Supplementary Figure 5b present the approximated fiber bundle entrance plane used for fitting purposes.

Single photon avalanche detectors (SPADs) differ in detection efficiencies and dark current levels. In order to take that into account in the localization process we performed reference measurements for both specifications. SPAD dark current was estimated by averaging over a several minute exposure time in complete darkness conditions. Detections efficiency variation between the detectors was measured by a long exposure to a smooth feature in a film of fluorescent dye. The detection rate differences between individual fibers were as high as 30% although a reference image of the same area showed less than 1% variance in brightness. A thorough description of the implementation of detector efficiencies and dark current in the localization algorithm is given in Supplementary Note 2.
